# Expression of HSP47 in Usual Interstitial Pneumonia and Nonspecific Interstitial Pneumonia

**DOI:** 10.1186/1465-9921-6-57

**Published:** 2005-06-14

**Authors:** Tomoyuki Kakugawa, Hiroshi Mukae, Tomayoshi Hayashi, Hiroshi Ishii, Seiko Nakayama, Noriho Sakamoto, Sumako Yoshioka, Kanako Sugiyama, Mariko Mine, Yohei Mizuta, Shigeru Kohno

**Affiliations:** 1 Second Department of Internal Medicine, Nagasaki University School of Medicine, Nagasaki, Japan; 2 Department of Pathology, Nagasaki University Hospital, Nagasaki, Japan; 3 Biostatistics Section, Division of Scientific Data Registry, Atomic Bomb Disease Institute, Nagasaki University Graduate School of Biomedical Sciences, Nagasaki; Japan

**Keywords:** usual interstitial pneumonia, nonspecific interstitial pneumonia, collagen vascular disease, heat shock protein 47, type I procollagens

## Abstract

**Background:**

Heat shock protein (HSP) 47, a collagen-specific molecular chaperone, is involved in the processing and/or secretion of procollagens, and its expression is increased in various fibrotic diseases. The aim of this study was to determine whether quantitative immunohistochemical evaluation of the expression levels of HSP47, type I procollagen and α-smooth muscle actin (SMA) allows the differentiation of idiopathic usual interstitial pneumonia (UIP) from UIP associated with collagen vascular disease (CVD) and idiopathic nonspecific interstitial pneumonia (NSIP).

**Methods:**

We reviewed surgical lung biopsy specimens of 19 patients with idiopathic UIP, 7 with CVD-associated UIP and 16 with idiopathic NSIP and assigned a score for the expression of HSP47, type I procollagen and α-SMA in type II pneumocytes and/or lung fibroblasts (score 0 = no; 1 = weak; 2 = moderate; 3 = strong staining).

**Results:**

The expression level of HSP47 in type II pneumocytes of idiopathic UIP was significantly higher than in CVD-associated UIP and idiopathic NSIP. The expression of HSP47 in fibroblasts was significantly higher in idiopathic UIP and idiopathic NSIP than in CVD-associated UIP. The expression of type I procollagen in type II pneumocytes was significantly higher in idiopathic UIP than in idiopathic NSIP. The expression of type I procollagen in fibroblasts was not different in the three groups, while the expression of α-SMA in fibroblasts was significantly higher in idiopathic UIP than in idiopathic NSIP.

**Conclusion:**

Our results suggest the existence of different fibrotic pathways among these groups involved in the expression of HSP47 and type I procollagen.

## Background

Heat shock protein (HSP) 47 is a collagen-binding, stress-inducible protein localized in the endoplasmic reticulum and is never released into the extracellular matrix. HSP47 plays a specific role in the intracellular processing of procollagen production as a collagen-specific molecular chaperone [1-4]. HSP47 expression has been shown to be upregulated in experimental animal models of fibrosis, including murine bleomycin-induced pulmonary fibrosis [5,6], rat peritoneal sclerosis [7] and carbon tetrachloride-induced rat liver cirrhosis [8]. In addition, we previously reported increased expression of human HSP47 in the fibrotic lesions of idiopathic pulmonary fibrosis (IPF) [9], fibrotic transplanted kidney [10], and peritoneal sclerosis [11]. Recent reports have demonstrated that HSP47 expression is highly tissue- and cell-specific, and is restricted to most phenotypically altered collagen-producing cells, and correlates well with that of collagen [9-11]. These findings suggest the important role of HSP47 in collagen synthesis in various fibrotic disorders. Furthermore, it was demonstrated that inhibition of HSP47 by antisense oligodeoxynucleotides markedly suppressed the production of collagen in 3T6 cells [4], in experimental proliferative glomerulonephritis [12] and in experimental peritoneal fibrosis [7]. These findings suggest that HSP47 might be a promising target for the treatment of fibrotic diseases.

Increased numbers of myofibroblasts in pulmonary fibrosis has been documented in both human lung tissues and those of animal models [5,6,13,14]. Myofibroblasts participate in remodeling and progressive destruction of the lung parenchyma through several mechanisms including increased extracellular matrix synthesis and contractility of the lung parenchyma [15]. We also showed previously that bleomycin treatment induced a marked increase in myofibroblasts in the active fibrotic areas of the lung from the early fibrotic stage in mice [5,6]. Activation of alpha-smooth muscle actin (α-SMA)-positive myofibroblasts is believed to play a key role in the progression of pulmonary fibrosis.

Patients with usual interstitial pneumonia (UIP) associated with a collagen vascular disease (CVD) have an improved prognosis compared with patients with idiopathic UIP [16]. Therefore, whether CVD-associated UIP and idiopathic UIP are the same pathological entity remains unresolved. Although identifying the pattern of UIP on surgical lung biopsy provides important prognostic data in distinction from other patterns of interstitial pneumonia [17-19], there are few studies that analyzed the individual histopathologic features comprising this pattern [20]. Although Flaherty *et al *[16] recently reported that patients with CVD-associated UIP have fewer fibroblastic foci in the lung and better prognosis than those with idiopathic UIP, the phenotypic difference of lung cells (e.g., fibroblasts and alveolar epithelial cells) between these two diseases and its association with the prognosis are poorly understood.

The recognition that lung biopsy samples of some patients with idiopathic interstitial disease do not fit into any well-defined histopathologic patterns of idiopathic interstitial pneumonia led to the proposal of the terms "unclassified interstitial pneumonia" by Kitaichi in 1990 [21] and nonspecific interstitial pneumonia (NSIP) by Katzenstein and Fiorelli in 1994 [22]. NSIP is characterized by varying degrees of inflammation and fibrosis within the alveolar walls. However, the most distinctive feature of NSIP is the temporal uniformity, which is in sharp contrast to the temporal heterogeneity seen in UIP. Among idiopathic interstitial pneumonias, NSIP has a more favorable response to corticosteroids and a better prognosis than UIP [17-19,23,24]. Based on these studies, NSIP is currently accepted as a clinicopathological entity in idiopathic interstitial pneumonias. Based on this background, we speculated that the pathogenesis of NSIP is different from UIP. In this regard, the phenotypic difference in fibroblasts and epithelial cells between these diseases is also not well elucidated.

The main hypothesis of the present study was that the expression levels of HSP47 in alveolar epithelial cells and lung fibroblasts are different in idiopathic UIP, CVD-associated UIP and idiopathic NSIP, thus allowing differentiation of these conditions. To test our hypothesis, we determined the expression levels of HSP47, type I procollagen and α-SMA in fibroblasts and type II pneumocytes in surgical lung biopsy specimens by immunohistochemistry.

## Materials and methods

### Study populations

The subjects of this study were patients enrolled in the hospitals of Nagasaki University School of Medicine. The study protocol was approved by the institutional review board, and informed consent was obtained from each patient. They included 19 patients with idiopathic UIP, 7 with CVD-associated UIP and 16 with idiopathic NSIP. Patients with idiopathic UIP and idiopathic NSIP had neither shown any signs nor positive serological and other markers of CVD. The associated diagnosis in the CVD-associated UIP patients were systemic sclerosis (n = 3), systemic sclerosis with Sjögren syndrome (n = 1), polymyositis with Sjögren syndrome (n = 1), primary Sjögren syndrome (n = 1) and mixed connective tissue disease (n = 1). Idiopathic NSIP patients included 8 with cellular and fibrosing pattern and 8 with fibrosing pattern. None of these patients had received steroids or other immunosuppressants therapy at the time of clinical sample collection. Patients characteristics before lung biopsy including age, smoking history, period from the onset to the surgical lung biopsy, results of pulmonary function tests and arterial blood gas analysis were collected from either the hospital medical records or those of the general practitioners. In the patients in this study, a suspicion of interstitial pneumonia was based on symptoms, physiologic abnormalities, and HRCT findings. The diagnosis was pathologically confirmed by open lung biopsy or video-assisted thoracoscopic surgery from multiple lobes in all patients and classified according to the American Thoracic Society/European Respiratory Society consensus criteria [25]. Control lung tissues were obtained from normal areas of lungs surgically removed for lung cancer (11 men and 9 women; median age, 62 years; range, 47 to 81 years).

### Antibodies

The primary antibodies used for the immunohistochemical studies included anti-human procollagen type I (Chemicon, Temecula, CA), anti-α-SMA (Neomarkers, Fremont, CA), anti-human cytokeratin (clone MNF116; Dako Corporation, Carpinteria, CA) and anti-HSP47 (Biotechnologies Corp., Victoria, BC, Canada). α-SMA and cytokeratin were used as markers of myofibroblasts and epithelial cells, respectively. Negative control studies were performed by using irrelevant immunoglobulin G with the same subclass of the first antibodies instead of the primary antibodies.

### Immunohistochemistry

Immunohistochemistry was performed with the conventional avidin-biotin-peroxidase histochemical technique using Vecstain Elite ABC Kit (Vector Laboratories, Burlingame, CA). Briefly, sequential paraffin sections (4-μm-thick) were deparaffinized with toluene and rinsed thoroughly with ethanol. The sections were then soaked in 0.3% H_2_O_2 _with absolute methanol for 20 minutes at room temperature to inactivate the endogenous peroxidase activity. They were incubated with blocking serum for 30 minutes, and then covered with primary antibodies and incubated for 1 hour. After washing in phosphate-buffered saline (PBS), the sections were processed further using the kits according to the instructions provided by the manufacturer, and then developed with 3,3'-diaminobenzidine and H_2_O_2_, followed by the Mayer's hematoxylin staining method.

### Pathologic Assessment

The profusion of fibroblastic foci in idiopathic UIP and CVD-associated UIP was graded semiquantitatively using a grading system of 0 to 3 (0 = absent; 1 = mild; 2 = moderate; 3 = marked) according to the method described previously [16]. The staining intensity and distribution of HSP47, α-SMA and type I procollagen in fibroblasts in active fibrotic areas (fibroblastic foci in UIP cases; alveolar septal interstitium expanded by fibrosis and intra-alveolar organizing fibrosis in NSIP cases) was also scored semiquantitatively using a grading system of 0 to 3 (0 = no staining; 1 = weak staining; 2 = moderate staining; 3 = strong staining). The expression levels of HSP47 and type I procollagen in type II pneumocytes were scored in a similar manner. The fibroblastic foci score and immunohistochemical score for each patient was calculated by averaging the score of each lobe. Histological sections were assessed independently, twice by each of two observers who were blind to the groups. The results were reproducible for interobserver and intraobserver variability. Statistical analysis was applied to representative results of one observer.

### Statistical Analysis

All values were expressed as median (range). Differences in categorical data between two groups were assessed by the Fisher's exact probability test for data of two categories and by chi-square test for those of three or more categories. Differences in continuous data between two groups were assessed by using the Wilcoxon rank-sum test and among three groups by the Kruskal-Wallis rank test. In addition, logistic regression was used to examine the relationships between the presence/absence of CVD-associated UIP, idiopathic NSIP and other variables. Statistical significance was defined by a *p *value of less than 0.05. Statistical analyses were carried out using SAS (SAS Institute, Inc., Cary, NC).

## Results

### Patient Characteristics

Table 1 shows the characteristics of patients enrolled in this study. Baseline demographic and physiologic characteristics were similar among the groups, except for sex and percentage of predicted total lung capacity.

### Histopathological and Immunohistochemical Findings

**Figure 1:**
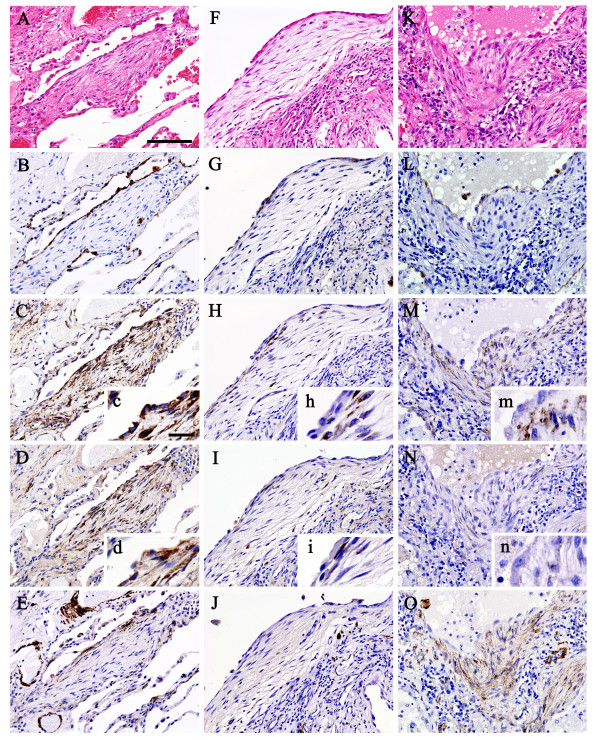
Photomicrographs of histopathological and immunohistochemical studies of representative surgical lung biopsy specimens (A-E; idiopathic UIP, F-J; CVD-associated UIP, K-O; idiopathic NSIP, scale bar = 100 μm). Histopathological examination (hematoxylin-eosin staining) revealed fibroblastic foci in both idiopathic UIP (A) and CVD-associated UIP (F), and fibroblast proliferation in idiopathic NSIP (K). Hyperplastic cuboidal epithelial cells were stained with cytokeratin, indicating that these cells were type II pneumocytes (B, G and L). Strong expression of HSP47 was noted predominantly in fibroblasts and type II pneumocytes in idiopathic UIP (C). Weak or no expression of HSP47 was noted in fibroblasts and type II pneumocytes in CVD-associated UIP (H). In idiopathic NSIP, strong expression of HSP47 was noted in fibroblasts, but not in type II pneumocytes (M). Type I procollagen was strongly expressed predominantly in fibroblasts and type II pneumocytes in idiopathic UIP (D), but neither in CVD-associated UIP (I) nor idiopathic NSIP (N). Expression of α-SMA was noted in some of fibroblasts, indicating that these cells were myofibroblasts, in all three diseases (E, J and O). Insets c, d, h, i, m and n are pictures taken at high power magnification (scale bar = 20 μm) of corresponding C, D, H, I, M and N sections to clearly show the phenotypic difference of type II pneumocytes. α-SMA = α-smooth muscle actin; CVD = collagen vascular disease; HSP47 = heat shock protein 47; NSIP = nonspecific interstitial pneumonia; UIP = usual interstitial pneumonia.

Photomicrographs of histological and immunohistochemical studies of representative surgical lung biopsy specimens are shown in Figure 1 (A-E; idiopathic UIP, F-J; CVD-associated UIP, K-O; idiopathic NSIP). Figure 1A–E, F–J and 1K–O represent sequential sections, respectively. Histopathological examination revealed fibroblastic foci in both idiopathic UIP (Fig. 1A) and CVD-associated UIP (Fig. 1F) and fibroblast proliferation in idiopathic NSIP (Fig. 1K). In all diseases, distinct aggregates of closely spaced fibroblasts with little intervening collagen deposition were seen and hyperplastic alveolar lining cells covered their luminal surface. Cuboidal epithelial cells were stained with cytokeratin, indicating that these cells were type II pneumocytes (Fig. 1B, G and 1L). Strong expression of HSP47 was noted predominantly in fibroblasts and most of type II pneumocytes in idiopathic UIP (Fig. 1C). In contrast, weak or no expression of HSP47 was noted in fibroblasts and type II pneumocytes in CVD-associated UIP (Fig. 1H). In idiopathic NSIP, strong expression of HSP47 was noted in fibroblasts, but not in type II pneumocytes (Fig. 1M). Type I procollagen was strongly expressed predominantly in fibroblasts and most of type II pneumocytes in idiopathic UIP (Fig. 1D), but neither in CVD-associated UIP (Fig. 1I) nor idiopathic NSIP (Fig. 1N). Expression of α-SMA was noted in some of fibroblasts in all three diseases, indicating that these cells were myofibroblasts (Fig. 1E, J and 1O). Negative control studies using non-specific immunoglobulin-G revealed no positive cells (data not shown).

**Table 1:** Patient Characteristics

	Idiopathic UIP	CVD-associated UIP	Idiopathic NSIP	p value
Age (years)	64 (34–72)	44 (38–63)	58 (28–75)	0.06
Sex (male/female)	17/2	1/6	5/11	0.0002
Smoking (none/ex/smoke)	6/7/6	5/2/0	10/3/2	0.15
Symptom onset (months)	9 (5–38)	12 (4–35)	6 (1–74)	0.44
Spirometry:				
VC (L)	2.63 (1.28–3.81)	2.01 (1.34–2.63)	2.50 (1.11–3.39)	0.10
predicted VC (%)	78.6 (55.8–117.0)	67.8 (53.5–112.3)	79.9 (50.0–108.1)	0.59
FEV1 (L)	2.18 (1.08–2.81)	1.67 (1.13–2.28)	1.72 (0.99–2.98)	0.05
predicted FEV (%)	84.10 (65.5–90.6)	84.3 (78.1–94.6)	80.79 (51.0–96.2)	0.19
Gas exchange:				
DLco (ml/min/mmHg)	9.13 (6.5–14.65)	9.94 (3.96–20.27)	11.45 (6.0–19.85)	0.35
predicted DLco (%)	49.00 (37.2–97.3)	43.80 (25.2–61.4)	57.85 (44.7–109.3)	0.05
Lung volume:				
predicted TLC (%)	62.45 (45.2–96.7)	79.90 (58.9–107.6)	71.55 (62.9–112.4)	0.04
TLC (L)	3.52 (1.78–5.39)	3.07 (2.0–4.0)	3.83 (1.8–8.8)	0.41
Arterial blood gases:				
PaO_2 _(mmHg)	82.5 (58.3–108.0)	89.2 (40.5–97.5)	83.4 (67.0–92.9)	0.43

Data are median (range).

α-SMA = α-smooth muscle actin; CVD = collagen vascular disease; DLco = diffusing capacity for carbon monoxide; HSP47 = heat shock protein 47; NSIP = nonspecific interstitial pneumonia; Symptom onset = period from the onset to the surgical lung biopsy; TLC = total lung capacity; UIP = usual interstitial pneumonia; VC = vital capacity.

### Results of Pathologic Assessment

**Figure 2:**
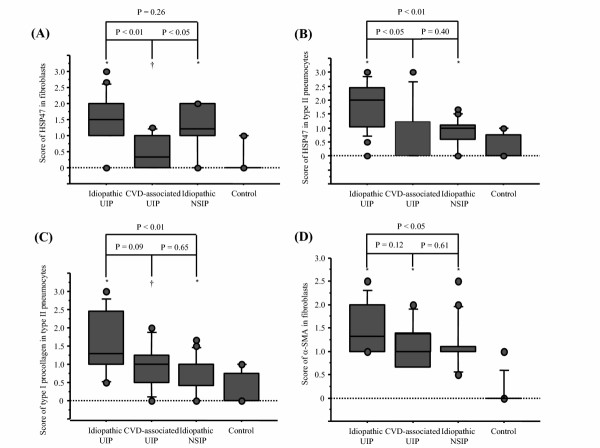
Box-and-whisker plots of HSP47, type I procollagen and α-SMA in patients with idiopathic UIP, CVD-associated UIP and idiopathic NSIP. (A) Patients with idiopathic UIP and idiopathic NSIP (P < 0.05) had a significantly higher expression of HSP47 in fibroblasts than those with CVD-associated UIP. (B) Expression of HSP47 in type II pneumocytes of patients with idiopathic UIP was significantly higher than that of CVD-associated UIP and idiopathic NSIP. (C) Expression of type I procollagen in type II pneumocytes of patients with idiopathic UIP was significantly higher than in idiopathic NSIP. (D) Patients with idiopathic UIP had a significantly higher expression of α-SMA in fibroblasts than those with idiopathic NSIP. *P < 0.01, compared with control. †P < 0.05, compared with control. The boxes represent the 25th to 75th percentiles, the solid lines within the boxes show the median values, the whiskers are the 10th and 90th percentiles, and the points represent outliers. For abbreviations, see Figure 1.

Patients with idiopathic UIP had higher profusion of fibroblastic foci (median, 2.0 [range, 0.67–3]) than those with CVD-associated UIP (1.0 [0–2]), albeit non-significantly (p = 0.06). Patients with idiopathic UIP (P < 0.01), CVD-associated UIP (P < 0.05) and idiopathic NSIP (P < 0.01) had a significantly higher expression of HSP47 in fibroblasts than that in control. Patients with idiopathic UIP (P < 0.01) and idiopathic NSIP (P < 0.05) had a significantly higher expression of HSP47 in fibroblasts than that in CVD-associated UIP (Fig. 2A). Patients with idiopathic UIP (P < 0.01) and idiopathic NSIP (P < 0.01) had a significantly higher expression of HSP47 in type II pneumocytes than that in control, while there was no significant difference between CVD-associated UIP and control. Expression of HSP47 in type II pneumocytes of patients with idiopathic UIP was significantly higher than those of CVD-associated UIP (P < 0.05) and idiopathic NSIP (P < 0.01, Fig. 2B). Patients with idiopathic UIP (P < 0.01) and idiopathic NSIP (P < 0.05) had a significantly higher expression of type I procollagen in fibroblasts compared with control, while there was no significant difference between CVD-associated UIP and control (1.0 [0–3], 1.0 [0–1.5], 1.0 [0–1.5] and 0.0 [0–1] for idiopathic UIP, CVD-associated UIP, idiopathic NSIP and control, respectively). Patients with idiopathic UIP (P < 0.01), CVD-associated UIP (P < 0.05) and idiopathic NSIP (P < 0.01) had a significantly higher expression of type I procollagen in type II pneumocytes than that in control (Fig. 2C). There was no significant difference in the expression of type I procollagen in fibroblasts among idiopathic UIP, CVD-associated UIP and idiopathic NSIP, while that in type II pneumocytes in patients with idiopathic UIP was significantly higher than in idiopathic NSIP (P < 0.01, Fig. 2C). Patients with idiopathic UIP (P < 0.01), CVD-associated UIP (P < 0.01) and idiopathic NSIP (P < 0.01) had a significantly higher expression of α-SMA in fibroblasts than that in control (Fig. 2D). Patients with idiopathic UIP had a significantly higher expression of α-SMA in fibroblasts than those with idiopathic NSIP (P < 0.05, Fig. 2D).

We further examined the differences between groups by univariate analysis based on the results of the pathological assessment (Table 2). The expression of HSP47 in fibroblasts was the most discriminative baseline feature for separating idiopathic UIP from CVD-associated UIP. The odds ratio of having idiopathic UIP compared with CVD-associated UIP was 12.68 for a unit increase in the mean score of the expression of HSP47 in fibroblasts. The expression of HSP47 in type II pneumocytes was the most discriminative baseline feature for separating idiopathic UIP from idiopathic NSIP. The odds ratio of having idiopathic UIP compared with idiopathic NSIP was 6.66 for a unit increase in the mean score of the expression of HSP47 in type II pneumocytes.

**Table 2:** Univariate Analysis of the Results of Pathologic Assessment.

Predictor	Odds Ratio	95% confidence interval	p Value
Idiopathic UIP vs CVD-associated UIP			
Fibroblastic foci score	3.73	0.94–14.79	0.06
HSP47 in fibroblasts	12.68	1.53–105.19	0.02
HSP47 in type II pneumocytes	3.39	1.10–10.45	0.03
α-SMA in fibroblasts	4.78	0.44–51.27	0.20
type I procollagen in fibroblasts	2.73	0.61–12.23	0.19
type I procollagen in type II pneumocytes	3.43	0.77–15.32	0.11
Idiopathic UIP vs Idiopathic NSIP			
HSP47 in fibroblasts	1.91	0.74–4.95	0.17
HSP47 in type II pneumocytes	6.66	1.76–25.15	0.01
α-SMA in fibroblasts	4.46	0.94–21.17	0.06
type I procollagen in fibroblasts	3.60	0.94–13.77	0.06
type I procollagen in type II pneumocytes	6.10	1.43–25.93	0.01

For abbreviations, see Table 1.

## Discussion

It is important to know the cell type(s) that contributes to lung fibrogenesis. The histopathologic pattern of UIP is well-characterized, with the cardinal feature being the patchy distribution of temporally heterogeneous fibrosis, comprising areas of established fibrosis with adjacent foci of fibroblastic proliferation, so-called fibroblastic foci. Recent studies have emphasized the importance of the fibroblastic focus, a manifestation of ongoing lung injury in patients with established fibrosis [13,15,26-29]. A number of investigators have stressed the prognostic value of quantifying fibroblastic foci in patients with idiopathic interstitial pneumonia [28,29]. Furthermore, a previous study showed that patients with CVD-associated UIP have fewer fibroblastic foci and better prognosis compared to patients with idiopathic UIP [16]. In idiopathic NSIP, which exhibits a more favorable response to corticosteroids and has a better prognosis than idiopathic UIP [17-19,23,24], fibroblastic foci with dense fibrosis are inconspicuous or absent [25]. Thus, fibroblasts are believed to play an important role in the progression of chronic pulmonary fibrosis. However, previous studies did not fully elucidate the phenotypic differences in fibroblasts among idiopathic UIP, CVD-associated UIP and idiopathic NSIP.

We identified higher expression of HSP47 in fibroblasts in patients with idiopathic UIP and idiopathic NSIP compared with CVD-associated UIP. We also showed that patients with idiopathic UIP had a significantly higher expression of α-SMA in fibroblasts than those with idiopathic NSIP. The differential expression of HSP47 and α-SMA in fibroblasts suggests that the underlying pathogenic process of fibrosis in these diseases may be distinctly different. The results emphasized that the expression of HSP47 in fibroblasts is the most discriminative features between idiopathic UIP and CVD-associated UIP, as identified by logistic regression. These findings imply that the expression of HSP47 in fibroblasts could also have important prognostic implications in interstitial pneumonias.

Our results also emphasized the importance of the phenotypic difference of epithelial cells among idiopathic UIP, CVD-associated UIP and idiopathic NSIP. Type II pneumocytes hyperplasia in areas of inflammation and fibrosis is commonly seen in both UIP and NSIP [25]. Although several studies performed in experimental models emphasized the importance of the alveolar epithelium in normal repair [30,31], the prevailing hypothesis regarding the pathogenesis of interstitial pulmonary fibrosis has been that the disease is due to a chronic unsolved inflammatory response, and in this context, the possible role of the epithelium has been largely neglected. However, there is increasing evidence supporting the notion that epithelial injury in the absence of ongoing inflammation is sufficient to cause the development of pulmonary fibrosis [15,30,31] and that alveolar epithelial cells are the primary source of cytokines and growth factors involved in fibroblast migration and proliferation [15]. In spite of these findings, the precise biological link between alveolar epithelial injury and fibrosis represent a challenging puzzle in which several pieces remain to be assembled.

We recently showed in rodent bleomycin-induced pulmonary fibrosis model that type II pneumocytes start to express HSP47 with the progression of fibrosis [5,6]. The present study extended this finding by showing that hyperplastic type II pneumocytes in idiopathic UIP express both HSP47 and type I procollagen. These findings suggest that increased number of type II pneumocytes in addition to fibroblasts produce type I procollagen through the induction of HSP47 and play an important role in the development of fibrosis. Our study also demonstrates a lower expression of HSP47 and type I procollagen in type II pneumocytes in CVD-associated UIP and idiopathic NSIP compared with idiopathic UIP. Interestingly, the expression level of HSP47 in type II pneumocytes was much higher in idiopathic UIP than in idiopathic NSIP, while there was no significant difference in the expression level of HSP47 in fibroblasts between them. Furthermore, the expression of type I procollagen in type II pneumocytes was different among idiopathic UIP, CVD-associated UIP and idiopathic NSIP in spite of the similar expression level of type I procollagen in fibroblasts. These findings suggest that the phenotypic difference of type II pneumocytes in interstitial pneumonias is more important biological feature than that of fibroblasts. In addition, we previously reported that treatment with pirfenidone, an anti-fibrotic agent, decreased HSP47-positive type II pneumocytes in bleomycin-induced pulmonary fibrosis in mice [6]. This finding further suggests that HSP47-positive type II pneumocytes might be promising target for therapeutic strategies designed for idiopathic UIP.

Several lines of evidence suggest that in some forms of tissue fibrosis, an epithelial-mesenchymal transition actively participates in the local formation of interstitial fibroblasts [32-34]. This process has been particularly studied in renal fibrosis. According to these findings, epithelial cells that detach from their basement membrane enter into epithelial-mesenchymal transition, express HSP47 and type I procollagen and divide as fibroblasts [32-34]. Whether this process takes place in the lung in IPF or in other forms of pulmonary fibrosis is largely unknown, but awaits further evaluation both *in vitro *and *in vivo*. The present findings that type II pneumocytes express HSP47 and type I procollagen in idiopathic UIP imply that a similar process could take place in idiopathic UIP. Further studies are warranted in order to elucidate the precise mechanism(s) involved in this process.

In this study, we also investigated whether the expression of HSP47, type I procollagen and α-SMA in fibroblasts and/or type II pneumocytes correlated with clinical course. However, no pathologic feature was associated with survival nor clinical data such as results of pulmonary function tests and arterial blood gas analysis (data not shown). This is probably because only small number of patients was assessed in this study. Further studies of a large number of patients are required.

## Conclusion

In summary, we have shown that type II pneumocytes and/or lung fibroblasts of patients with idiopathic UIP, CVD-associated UIP and idiopathic NSIP express different levels of HSP47 and type I procollagen. Our findings support the concept that these diseases are different pathophysiological entities with different fibrotic pathways. We speculate that careful immunohistochemical evaluation of HSP47, α-SMA and type I procollagen in the lung may be a useful method to understand differences in the underlying pathogenic mechanisms of these diseases. Further studies of a large number of patients are required to determine the prognostic and therapeutic values of HSP47 expression.

## Abbreviations

**α-SMA**: alpha-smooth muscle actin

**CVD**: collagen vascular disease

**HSP**: heat shock protein

**IPF**: idiopathic pulmonary fibrosis

**NSIP**: nonspecific interstitial pneumonia

**PBS**: phosphate-buffered saline

**UIP**: usual interstitial pneumonia

## Authours' contributions

TK, HM, TH and MM have made substantial contribution to acquisition and analysis of data.

TK, HM, TH and SK have made substantial contributions to conception and design.

TK, HM, TH and SK have been involved in drafting the article.

HI, SN, NS, SY, KS and YM have been involved in revising it critically for important intellectual content.
